# Association of Mechanical Circulatory Support With In‐Hospital Outcomes in Non‐Ischemic Cardiogenic Shock: A Nationwide Inpatient Study

**DOI:** 10.1002/clc.70416

**Published:** 2026-07-23

**Authors:** Li Liu, Burhan Memon, Tian Zou, Wiaam Elkhatib, Pincheng Luo, Laura E. Billstein, Nomesh Kumar, Youyou Cheng, Abigail Fickel, Shengtong Shao, Maya Guglin, Chenyu Sun

**Affiliations:** ^1^ The Second People's Hospital of Hefei Hefei Anhui China; ^2^ Hefei Hospital Affiliated to Anhui Medical University Hefei Anhui China; ^3^ OSF Saint Francis Medical Center Peoria Illinois USA; ^4^ Department of Cardiovascular Medicine Mayo Clinic Rochester Minnesota USA; ^5^ Department of Cardiovascular Diseases Mayo Clinic Jacksonville Florida USA; ^6^ School of Medicine University of Galway Galway Ireland; ^7^ Division of Public Health, Infectious Diseases, and Occupational Medicine, Mayo Clinic Rochester Minnesota USA; ^8^ Mayo Clinic School of Graduate Medical Education, Mayo Clinic College of Medicine and Science Rochester Minnesota USA; ^9^ School of Public Health University of Minnesota Twin Cities Minneapolis Minnesota USA; ^10^ University of the Incarnate Word School of Osteopathic Medicine San Antonio Texas USA; ^11^ Mayo Clinic Alix School of Medicine, Mayo Clinic Rochester Minnesota USA; ^12^ Department of Nutrition and Food Studies New York University New York New York USA; ^13^ Section of Heart Failure Robert Wood Johnson University Hospital New Brunswick New Jersey USA

**Keywords:** cardiogenic shock, extracorporeal membrane oxygenation, health care burden, Impella, intra‐aortic balloon pump, mechanical circulatory support, non‐ischemic

## Abstract

**Introduction:**

Cardiogenic shock (CS) carries high morbidity and mortality. However, evidence for mechanical circulatory support (MCS) in non‐ischemic CS remains limited, as prior trials have focused on infarct‐related shock.

**Methods:**

We performed a retrospective cross‐sectional study using the Nationwide Inpatient Sample (2017–2019), identifying hospitalizations with a principal diagnosis of CS and excluding acute myocardial infarction. Patients were categorized into mutually exclusive groups: no MCS, intra‐aortic balloon pump (IABP) only, Impella only, or extracorporeal membrane oxygenation (ECMO) only. Primary outcomes were in‐hospital mortality, length of stay (LOS), and total hospital cost. Survey‐weighted multivariable models adjusted for demographics, hospital characteristics, and comorbidity burden using Elixhauser and Charlson indices.

**Results:**

Among 104 045 hospitalizations, 6040 received MCS. IABP was most common, followed by Impella and ECMO. Among patients receiving a single MCS strategy, overall in‐hospital mortality was 29.5%., with lower mortality in the IABP group (19.9%) compared with Impella (37.4%) and ECMO (44.3%). After adjustment, IABP was associated with lower observed in‐hospital mortality versus no MCS (aOR 0.58, 95% CI 0.53–0.64; *p* < 0.01), whereas Impella and ECMO showed increased aOR. IABP and ECMO were associated with longer LOS, while Impella was not. All MCS strategies were associated with higher costs, with ECMO showing the greatest increase (cost ratio 3.30, 95% CI 3.12–3.50).

**Conclusion:**

In non‐ischemic CS, MCS strategies demonstrate distinct profiles of mortality and resource utilization. IABP was associated with lower observed in‐hospital mortality, whereas all MCS were linked to higher costs, highlighting heterogeneity in outcomes and the need for further research.

## Graphical abstract

Among US hospitalizations for non‐ischemic cardiogenic shock, MCS strategies were associated with distinct mortality and resource utilization profiles. IABP showed lower observed mortality, whereas ECMO and Impella showed higher mortality and resource use; these observational associations may reflect treatment selection and shock severity rather than device ineffectiveness.

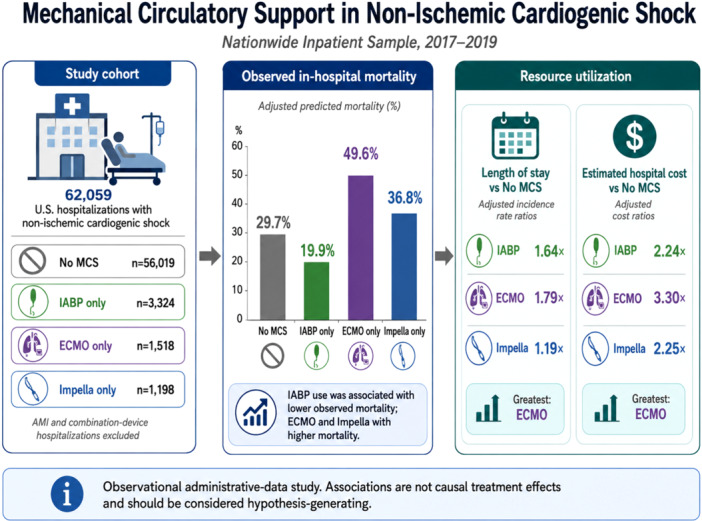

**Abbreviations**
AHRQ: Agency for Healthcare Research and Quality
AMI: acute myocardial infarction
CI: confidence interval
CS: cardiogenic shock
ECMO: extracorporeal membrane oxygenation
HCUP: Healthcare Cost and Utilization Project
HF: heart failure
IABP: intra‐aortic balloon pump
ICD‐10‐CM: International Classification of Diseases, 10th Revision, Clinical Modification
ICD‐10‐PCS: International Classification of Diseases, 10th Revision, Procedure Coding System
IQR: interquartile range
IRR: incidence Rate Ratio
LOS: length of stay
MCS: mechanical circulatory support
NIS: Nationwide Inpatient Sample
OR: odds ratio
SD: standard deviation
USD: United States Dollar
VA‐ECMO: veno‐arterial extracorporeal membrane oxygenation

## 1 Introduction

Cardiogenic shock (CS) remains the leading cause of death in patients hospitalized with acute cardiovascular conditions, carrying in‐hospital mortality rates of one quarter to about half of the patients with CS despite advances in revascularization and critical care [1, 2, 3]. While the epidemiology of CS has traditionally been dominated by acute myocardial infarction (AMI), recent data demonstrate that non‐AMI etiologies, primarily acute‐on‐chronic heart failure (HF) and de novo HF, now account for a substantial and growing proportion of CS cases [1, 3, 4]. In the multinational Critical Care Cardiology Trials Network (CCCTN) registry, HF‐related CS comprised nearly 60% of all CS admissions, with mortality of 25% even in acute‐on‐chronic HF related CS and 31% of de novo HF related CS [1].

Temporary mechanical circulatory support (MCS) has emerged as a cornerstone of CS management, offering the potential to stabilize hemodynamics, unload the failing ventricle, and preserve end‐organ perfusion [5]. However, the evidence guiding MCS deployment derives largely from studies of infarct‐related shock [6, 7]. The IABP‐SHOCK II trial demonstrated no mortality benefit with intra‐aortic balloon pump (IABP) in AMI‐CS [8], while more recent network meta‐analyses suggest potential long‐term survival advantages with Impella in selected infarct populations, albeit at the cost of increased bleeding and vascular complications [9]. For non‐ischemic CS, the evidence is limited [5, 10].

This evidence gap carries profound clinical and policy implications. Non‐ischemic CS differs fundamentally from infarct‐related shock in pathophysiology, hemodynamic profile, and potential for myocardial recovery [11, 12]. Yet current practice patterns and device selection in non‐ischemic CS remain guided largely by evidence from infarct populations, consensus opinion, clinical judgment, and institutional preference rather than high‐quality outcome data directly from non‐ischemic CS patients [5, 6, 9, 10, 12].

Furthermore, the economic implications of MCS deployment are substantial [13]. Understanding the relationship between MCS strategy, clinical outcomes, and resource utilization in non‐ischemic CS is essential for informed clinical decision‐making and health policy. Using a nationally representative cohort of hospitalizations for non‐ischemic CS, we aimed to describe patterns of MCS use and to compare in‐hospital mortality, length of stay (LOS), and total hospital cost across different MCS strategies.

## 2 Methods

### 2.1 Data Source and Study Population

A retrospective cross‐sectional analysis was conducted using data from the Nationwide Inpatient Sample (NIS) for the years 2017−2019. The NIS, developed by the Agency for Healthcare Research and Quality (AHRQ) as part of the Healthcare Cost and Utilization Project (HCUP), is the largest publicly available all‐payer inpatient healthcare database in the United States [14]. This study was deemed exempt from institutional review board approval as it uses publicly available, de‐identified data [15].

Hospitalizations for CS were identified using ICD‐10‐CM diagnosis code R57.0 in any diagnosis position. To isolate non‐ischemic etiologies, hospitalizations with concomitant diagnosis codes for AMI (ICD‐10‐CM I21.x, I22.x) were excluded.

### 2.2 Exposure and Outcomes

MCS device use was identified using ICD‐10‐PCS procedure codes: IABP (5A02210), ECMO (5A15223, 5A1522F, 5A1522G, and 5A1522H), and Impella (5A0221D, 5A0211D). Hospitalizations were categorized into mutually exclusive groups: (1) no MCS, (2) IABP only, (3) ECMO only, (4) Impella only. Combination groups were excluded to follow the HCUP rule to reduce the risk of individual identification of persons if any given cell of tabulated data is less than or equal to 10 [16].

All three outcomes were designated as primary: (1) in‐hospital mortality, defined as death during the index hospitalization; (2) hospital length of stay (LOS, in days); and (3) total hospital costs (calculated using the HCUP NIS cost‐to‐charge ratio and inflation‐adjusted to 2019 US dollars based on www.bls.gov/data/inflation_calculator.htm).

### 2.3 Covariates

Demographic characteristics including age and sex were extracted. To minimize the risk of individual identification [16], race/ethnicity, socioeconomic variables (median household income quartile based on patient ZIP code and primary payer), and hospital characteristics (teaching status [rural, urban non‐teaching, urban teaching], geographic region [Northeast, Midwest, South, West], and urban–rural location) were not fully analyzed for baseline description. Comorbidity burden was adjusted using the Elixhauser Comorbidity Index, which is designed for risk adjustment in administrative inpatient datasets and captures a broad range of pre‐existing conditions, including hypertension, diabetes mellitus, renal failure, chronic pulmonary disease, obesity, liver disease, and other clinically relevant comorbidities [17, 18] Individual Elixhauser component comorbidities were not entered simultaneously with the summary Elixhauser measure to avoid duplicative adjustment, collinearity, and overadjustment.

### 2.4 Statistical Analysis

Baseline characteristics were compared across MCS groups using ANOVA (continuous variables), survey‐weighted Pearson chi‐square tests (categorical variables), and Kruskal‐Wallis tests (skewed variables). Normally distributed continuous variables (age, Elixhauser, Charlson) are presented as means (SD), and skewed variables (LOS, cost) as medians (IQR). Missing or unknown covariate values were retained as separate categories; records with missing outcomes were excluded from the respective outcome‐specific analyses. Survey commands (svy) in STATA were used to account for the complex sampling design, including stratification, clustering, and discharge‐level weights. These design elements were incorporated in all regression and covariate‐adjusted analyses to generate national estimates. Multivariable logistic regression was used to examine the association with in‐hospital mortality, with results reported as odds ratios (ORs) and 95% confidence intervals (CIs). LOS was modeled using negative binomial regression due to overdispersion in the Poisson model (Pearson dispersion parameter ≈16.14). The estimated dispersion parameter was *α* = 0.80 (95% CI, 0.79–0.82). Results are reported as incidence rate ratios (IRRs). Total costs were analyzed using gamma regression with a log link to account for right‐skewed distributions, with results reported as cost ratios.

The primary multivariable model adjusted for age, sex, race, ZIP code income quartile, hospital teaching status, hospital region, urban–rural location, primary payer, and the Elixhauser Comorbidity Index [17, 18]. A sensitivity analysis substituted the Charlson Comorbidity Index [19, 20, 21] for Elixhauser to assess robustness. All statistical tests were 2‐sided, with *p* < 0.05 considered statistically significant. R version 4.5.2 (R Foundation for Statistical Computing, Vienna, Austria) was used for data visualization, including the construction of forest plots. All other analyses were performed using Stata version 17.0 (StataCorp LLC, College Station, Texas).

## 3 Results

### 3.1 Study Population and MCS Utilization

**Figure 1:**
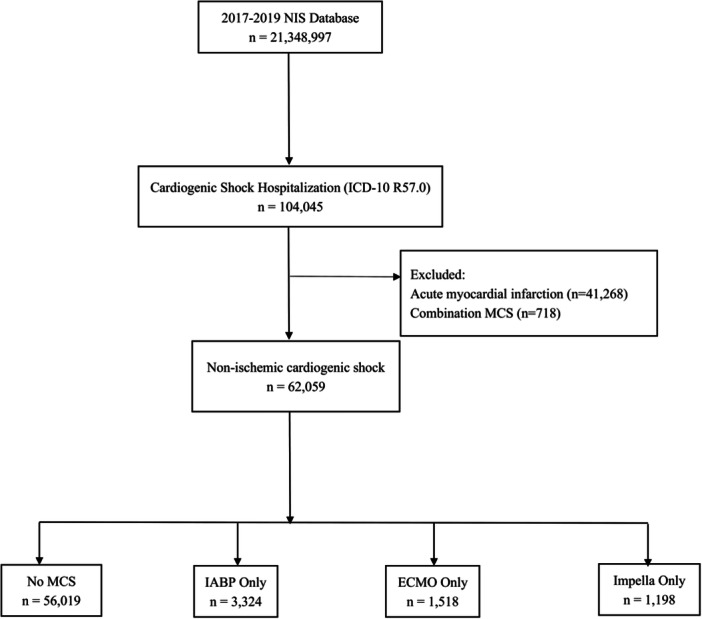
Flow chart of sample selection.

From 21 348 977 hospitalizations in the 2017–2019 NIS sample, 104 045 hospitalizations with a diagnosis of CS were identified. After excluding 41 268 with AMI records and 718 records with two ore three MCS strategies, the final analytic cohort comprised 62 059 hospitalizations for non‐ischemic CS. The study flow diagram is presented in Figure 1. The majority of hospitalizations (*n* = 56 019) did not receive MCS. Among those receiving MCS, IABP was the most common single device (3324), followed by Impella (1518) and ECMO (1198).

### 3.2 Baseline Characteristics

Table 1 presents baseline characteristics stratified by MCS strategy. The overall cohort had a mean age of 64.01 ± 17.45 years, with 23,856 (38.44%) females. Significant age differences were observed across MCS groups. ECMO‐only hospitalizations involved substantially younger individuals (mean age 44.34 ± 23.78 years) compared with other groups (*p* < 0.01). Nearly all ECMO was conducted in urban teaching (97.50%), while 82.78% of all non‐ischemic CS hospitalizations were in these hospitals. The comorbidity burden varied across groups, with the lowest observed in the ECMO‐only group.

**Table 1:** Baseline characteristics and unadjusted clinical outcomes by mechanical circulatory support strategy.

Characteristic	Total (*N* = 62 059)	No MCS (*n* = 56 019)	IABP only (*n* = 3324)	ECMO only (*n* = 1518)	Impella only (*n* = 1198)	*p* value
Age, years, mean ± SD	64.0 ± 17.5	64.7 ± 17.2	62.3 ± 13.3	44.3 ± 23.8	62.1 ± 14.3	**< 0.01**
Female, *n* (%)	23 856 (38.4)	21 887 (39.1)	1005 (30.2)	624 (41.1)	340 (28.4)	**< 0.01**
Elixhauser comorbidity sum, mean ± SD	6.5 ± 2.3	6.52 ± 2.3	6.8 ± 2.2	5.8 ± 2.4	6.6 ± 2.3	**< 0.01**
Charlson comorbidity index, mean ± SD	3.5 ± 2.3	3.6 ± 2.3	3.3 ± 2.0	2.2 ± 1.89	3.4 ± 2.1	**< 0.01**
In‐hospital mortality, *n* (%)	19 592 (31.6)	17 811 (31.8)	661 (19.9)	672 (44.3)	448 (37.4)	**< 0.01**
Length of stay, days, median [IQR]	8 [4–15]	8 [4–14]	14 [7–26]	17 [6–35]	9 [3–20]	**< 0.01**
Estimated hospital cost, 2019 USD, median [IQR]	$31 455 [$15 107–$65 436]	$28 194 [$14 076–$56 700]	$78 187 [$43 733–$153 273]	$155 225 [$81 792–$285 867]	$78 921 [$49 377–$133 410]	**< 0.01**

*Note: p* values represent overall comparisons across the four mutually exclusive MCS groups. Continuous normally distributed variables were compared using analysis of variance (ANOVA test); skewed variables were compared using the Kruskal−Wallis test. Categorical variables were compared using survey‐weighted Pearson chi‐square tests. Estimated hospital costs were calculated using the HCUP NIS cost‐to‐charge ratio and inflation‐adjusted to 2019 US dollars.

Abbreviations: ECMO, extracorporeal membrane oxygenation; IABP, intra‐aortic balloon pump; IQR, interquartile range; MCS, mechanical circulatory support; SD, standard deviation.

### 3.3 Unadjusted Outcomes

Table 1 also displays unadjusted clinical outcomes. Among the overall non‐ischemic CS cohort, in‐hospital mortality was 31.6%. Among hospitalizations receiving a single MCS strategy, overall in‐hospital mortality was 29.5%. Mortality varied across MCS strategies, with the lowest observed mortality in the IABP‐only group (19.9%), compared with ECMO‐only (44.3%) and Impella‐only (37.4%) hospitalizations.

**Figure 2:**
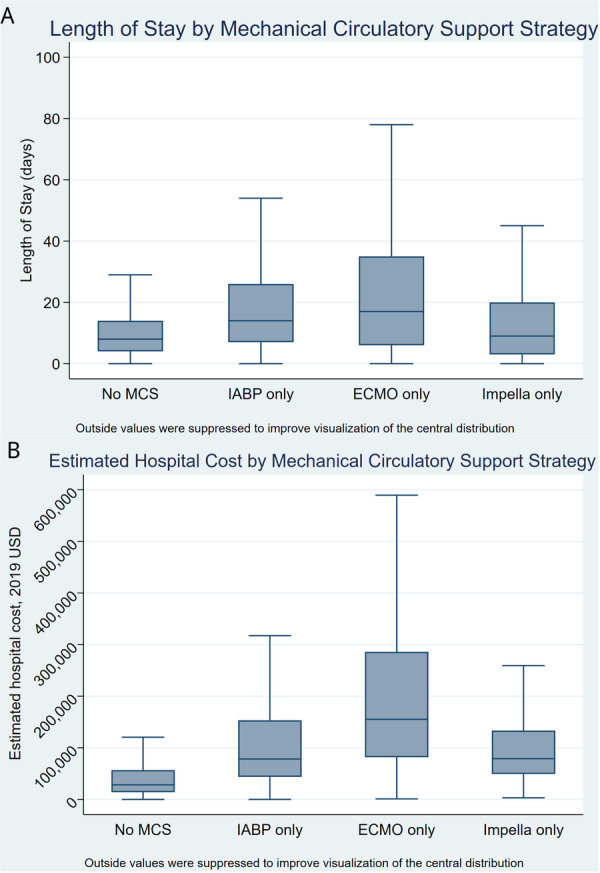
Lengths of stays (A) and total cost of hospitalization (B) by mechanical circulatory support types.

LOS also varied substantially (Figure 2A). Hospitalizations with Impella had the shortest median LOS (9 days, IQR 3–20) among those on MCS, and the ECMO group had longest LOS (median 17 days, IQR 6–35). Total hospital cost increased across MCS strategies (Figure 2B). Median costs were lowest in the no MCS group ($28 194, IQR $14 076–$56 700) and highest in the ECMO group ($155 225, IQR $81 792–$285 867). All MCS groups had substantially higher charges than the no MCS group (*p* < 0.01).

### 3.4 Correlation Between LOS and Total Costs

LOS and total costs were strongly correlated (Pearson's *r* = 0.7916, Spearman's ρ = 0.7911, both *p* < 0.001). (Supporting Information S1: Figure 1) In unadjusted survey‐weighted linear regression, each additional hospital day was associated with $4720 higher estimated hospital cost (95% CI, $4519–$4920; *p* < 0.01; *R* ^2^ = 0.627). After adjustment for demographics, hospital characteristics, payer, Elixhauser comorbidity burden, and MCS type, each additional hospital day remained associated with $4452 higher estimated hospital cost (95% CI, $4262–$4641; *p* < 0.01; *R* ^2^ = 0.683).

### 3.5 Adjusted Outcomes

**Figure 3:**
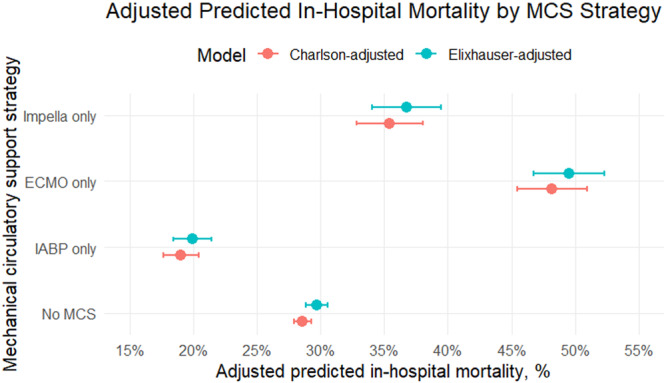
Foret plot of predicted in‐hospital mortality by different mechanical circulatory support types.

Table 2 presents adjusted ORs for in‐hospital mortality. IABP use was associated with significantly lower observed in‐hospital mortality (OR = 0.58, 95% CI 0.53–0.64, *p* < 0.01). In contrast, ECMO only (OR = 2.41, 95% CI 2.14–2.472, *p* < 0.01), and Impella only (OR = 1.39, 95% CI 1.24–1.57, *p* < 0.01) were associated with a statistically significant higher mortality compared with no MCS. Sensitivity analysis based on Charlson comorbidity index demonstrated similar results (Supporting Information S1: Table 1). Supporting Information S1: Table 2 and Figure 3 demonstrated the adjusted mortality rate.

**Table 2:** Elixhauser‐adjusted associations between mechanical circulatory support strategy and in‐hospital outcomes.

Outcome/MCS strategy	Ratio (95% CI)	*p* value
*In‐hospital mortality* a		
IABP only	0.58 (0.53–0.64)	< 0.01
ECMO only	2.41 (2.14–2.72)	< 0.01
Impella only	1.39 (1.24–1.57)	< 0.01
*Length of stay (days)* b		
IABP only	1.64 (1.57–1.70)	< 0.01
ECMO only	1.79 (1.68–1.91)	< 0.01
Impella only	1.19 (1.11–1.27)	< 0.01
*Estimated hospital cost, 2019 USD (USD)* c		
IABP only	2.24 (2.15–2.33)	< 0.01
ECMO only	3.30 (3.12–3.50)	< 0.01
Impella only	2.25 (2.13–2.38)	< 0.01

*Note:* Models adjusted for age, sex, race, ZIP income quartile, hospital teaching status, hospital region, urban‐rural location, primary payer, and Elixhauser comorbidity index. Bold indicates *p* < 0.05.

^a^ Adjusted odds ratio for in‐hospital mortality compared with no MCS.

^b^ Incidence rate ratios from negative binomial regression, representing ratio of expected LOS compared with No MCS.

^c^ Cost ratios from gamma regression with log link, representing ratio of expected cost (inflation‐adjusted to 2019 US dollars) compared with No MCS.

In the Elixhauser‐adjusted negative binomial regression model, all MCS strategies were associated with longer LOS compared with no MCS. The adjusted IRR for LOS were 1.64 for IABP (95% CI, 1.57–1.70; *p* < 0.01), 1.79 for ECMO (95% CI, 1.68–1.91; *p* < 0.01), and 1.19 for Impella (95% CI, 1.11–1.27; *p* < 0.01). In the Elixhauser‐adjusted gamma regression model with a log link, all MCS strategies were associated with higher inflation‐adjusted estimated hospital costs compared with no MCS. The adjusted cost ratios were 2.24 for IABP (95% CI, 2.15–2.33; *p* < 0.01), 3.30 for ECMO (95% CI, 3.12–3.50; *p* < 0.01), and 2.25 for Impella (95% CI, 2.13–2.38; *p* < 0.01). (Table 2) Sensitivity analysis based on Charlson comorbidity index demonstrated similar results (Supporting Information S1: Table 1).

## 4 Discussion

In this nationally representative cohort of hospitalizations for non‐ischemic CS, we found that MCS was deployed in approximately one in eight hospitalizations with a diagnosis of non‐ischemic CS, and IABP was the most commonly used device. Our principal finding is that MCS strategies were associated with distinct patterns of clinical outcomes and resource utilization in non‐ischemic CS. Compared with non‐MCS group, IABP use was associated with lower in‐hospital mortality, whereas Impella and ECMO were associated with significant higher mortality, longer hospitalizations and substantially higher costs. The consistency of our findings across two validated comorbidity measures strengthens confidence in the robustness of our adjustments [17, 18, 19, 20, 21].

Our observed overall mortality of 31. 6% is somewhat closer to but slightly lower than the CZECH‐SHOCK registry, a national prospective study of CS across all etiologies [2] likely reflecting our focus on non‐ischemic etiologies and the inclusion of patients managed without MCS who may have been considered either too ill or too stable for device deployment. Importantly, non‐ischemic CS represents a distinct clinical entity with different hemodynamic profiles, myocardial recovery potential, and therapeutic responsiveness compared with AMI‐related shock, which may partially account for these differences [11, 12]. The observed mortality signal associated with IABP, a 42% reduction in adjusted odds of death, stands in striking contrast to the neutral results of IABP‐SHOCK II in infarct‐related shock [8] This discrepancy underscores the limitation of extrapolating evidence from ischemic to non‐ischemic CS populations. In HF–related shock, afterload reduction and augmentation of coronary perfusion provided by IABP may be more physiologically aligned with the underlying disease process than in infarct‐related shock, where myocardial necrosis predominates [11, 12, 22] Furthermore, contemporary observational data continues to suggest that IABP may retain a role in selected CS phenotypes, particularly those with less severe hemodynamic compromise or greater likelihood of myocardial recovery [22, 23] Further study in non‐ischemic populations will be needed to affirm these findings in context of a recent 2025 RCT demonstrating a lack of survival benefit or bridging therapy at 60 day follow up in early IABP use for CS secondary to HF [24].

However, interpretation of this apparent mortality benefit must be cautious. A key consideration is confounding by indication, chronic comorbidity burden and shock severity. Although the IABP group in our cohort demonstrated higher comorbidity burden as measured by Charlson and Elixhauser indices, these indices primarily reflect chronic disease rather than acute hemodynamic derangement. Recent evidence demonstrates that comorbidity burden and acute shock severity represent distinct prognostic domains, with comorbidities exerting greater influence on outcomes in less severe shock stages [25, 26] while shock severity itself is a strong prognostic indicator [2] The observed lower mortality associated with IABP in the present study may therefore reflect selection of patients with less profound shock severity rather than a pure therapeutic effect. IABP also constituted the largest means of MCS in this patient sample, likely due to its superior afterload‐reducing effect in HF‐related CS compared to ischemic shock, fewer contraindications for use compared to ECMO or Impella, and overall favorable safety profile for use as a bridge to destination therapy [27]. Patients receiving more intensive MCS devices (ECMO, Impella) likely had more profound acute hemodynamic derangement despite similar or lower comorbidity scores, potentially explaining the absence of survival benefit in these groups. Additionally, LOS across hospitalizations were variable and remained relatively shorter compared to outcome data for larger trials focused on ischemic CS over 30‐day and 6‐month follow up periods. Future study of non‐ischemic CS over longer outcome durations may potentially yield greater insight into sustained MCS benefit or harm over time.

The lack of mortality benefit associated with Impella in our study is consistent with a growing body of literature. Randomized and observational comparisons in AMI‐CS have frequently demonstrated no survival advantage of Impella over IABP, despite more aggressive hemodynamic support [28, 29] Moreover, large contemporary registries have reported higher in‐hospital mortality and complication rates with Impella compared with IABP [30, 31, 32] Recent meta‐analyses further reinforce these findings, demonstrating no reduction or even an increase in short‐term mortality but significantly increased risks of major bleeding, vascular complications, and hemolysis with Impella [33, 34] While larger RCT's such as the landmark DanGer Shock trial [35] focused on ischemic CS and meta‐analysis of RCT's by Tariq et al. [36] have both demonstrated mortality benefit of Impella at the 6 month period, smaller RCT's including ISAR‐SHOCK and IMPRESS failed to demonstrate this benefit up to 5 years [37, 38].

Notably, even in non‐AMI CS populations, Impella has been associated with higher mortality compared with IABP [39] supporting the external validity of our findings. Similarly, in more advanced support scenarios such as VA‐ECMO, the addition of Impella for left ventricular unloading has not demonstrated survival benefit over IABP, while being associated with increased adverse events [40] In addition, a recent individual patient data meta‐analysis of four randomized clinical trials found no mortality benefit with the routine use of VA‑ECMO in AMI‑related CS, while its use was associated with higher rates of major bleeding and vascular complications [41] Collectively, these data suggest that escalation to more complex MCS devices does not necessarily translate into improved survival and may instead expose patients to additional procedural risks.

Several mechanisms may explain these observations. First, device‐related complications, including bleeding, vascular injury, hemolysis, and need for renal replacement therapy, are consistently higher with Impella and ECMO [9, 42]. These adverse events may offset the theoretical hemodynamic benefits of more advanced circulatory support. Second, the patients receiving these devices in our cohort differed systematically from those managed with IABP or medical therapy alone. The ECMO group was substantially younger, yet still experienced high mortality, suggesting that device selection may be reserved for the most critically ill patients in whom prognosis is already poor. While our multivariable models adjusted for measured confounders, residual confounding by CS severity likely persists, as administrative data lack granular hemodynamic metrics.

From a health systems perspective, our findings highlight the substantial resource utilization burden associated with MCS use. The strong correlation between LOS and total costs underscores the central role of ICU and hospital care duration in driving the economic burden of CS [13]. ECMO, in particular, was associated with a dramatic increase in cost, exceeding $155 225 in median cost and a more than threefold adjusted increase. This is consistent with prior comparative analyses demonstrating significantly higher resource utilization with ECMO compared with less invasive MCS strategies [43]. Importantly, our findings highlight that higher‐cost MCS strategies were not associated with lower observed in‐hospital mortality in this cohort, although these associations are likely influenced by differences in patient selection and shock severity. These results underscore the complexity of interpreting resource utilization in non‐ischemic CS.

This study has several important limitations. First, the observational design precludes causal inference, and residual confounding by shock severity and treatment selection is likely. Although we adjusted for demographics, hospital characteristics, payer, and comorbidity burden, the NIS does not capture key markers of acute shock severity, including lactate level, vasopressor use or dose, invasive hemodynamics, SOFA score components, SCAI shock stage, cardiac power output, or timing of MCS implantation. In addition, the timing of MCS placement relative to shock onset, cardiac arrest, organ failure, or escalation of care cannot be determined; therefore, early planned support cannot be distinguished from rescue or salvage support. Accordingly, the observed association between IABP use and lower in‐hospital mortality should be interpreted as hypothesis‐generating rather than causal. Second, the use of administrative data limits clinical granularity and may be subject to coding variability across institutions. Diagnosis and procedure coding practices may differ across hospitals, which could lead to misclassification of shock etiology, MCS use, comorbidities, or complications. Non‐ischemic CS is clinically heterogeneous, including de novo HF, acute‐on‐chronic HF, myocarditis, arrhythmia‐related shock, cardiomyopathy, valvular disease, and other etiologies; however, the NIS lacks the longitudinal clinical history, laboratory data, hemodynamic data, and advanced therapy evaluation information needed to reliably distinguish these phenotypes, particularly de novo versus acute‐on‐chronic HF‐related shock. Third, the HCUP cell size rules, while protecting patient privacy, further constrain subgroup analyses. Fourth, we were unable to assess post‐discharge outcomes, including long‐term survival, functional status, or readmission rates. Fifth, the current analysis was limited to the licensed datasets accessible to our research team at the time of analysis; therefore, only data from 2017 to 2019 were included. Finally, because the NIS does not capture the timing of diagnoses or procedures, acute in‐hospital events such as acute kidney injury, cardiac arrest, respiratory failure, intubation, stroke, major bleeding, acute liver injury, or tamponade could not be reliably distinguished as baseline severity markers, indications for MCS escalation, or post‐MCS complications. Therefore, these events were not analyzed as device‐associated secondary outcomes.

Despite these limitations, our study provides important real‐world evidence addressing a critical knowledge gap in non‐ischemic CS. The observed heterogeneity in outcomes across MCS strategies underscores the need for phenotype‐specific, prospective studies to guide device selection. Future research should focus on integrating hemodynamic profiling, shock staging, and timing of MCS deployment to optimize patient selection and improve outcomes in this increasingly prevalent population.

## 5 Conclusions

In this nationally representative cohort of hospitalizations for non‐ischemic CS, we observed that IABP use was associated with lower observed in‐hospital mortality, while all three MCS were associated with substantially higher costs, and both IABP and ECMO were associated with higher observed in‐hospital mortality, and longer hospitalizations. These findings underscore the need for research tailored to the growing non‐ischemic CS patient population to evaluate application of advanced MCS.

## Funding

The authors have nothing to report.

## Conflicts of Interest

The authors declare no conflicts of interest.

## Supporting information


Supporting File

## Data Availability

The data supporting the findings of this study are derived from the Nationwide Inpatient Sample (NIS) from the Healthcare Cost and Utilization Project (HCUP), Agency for Healthcare Research and Quality. Restrictions apply to the availability of these data, which were used under license for this study. NIS data are available to researchers upon completion of a data use agreement and payment of a data access fee through the HCUP Central Distributor (https://www.hcup‐us.ahrq.gov/).
