# Supplementary material for: Association of Mechanical Circulatory Support With In‐Hospital Outcomes in Non‐Ischemic Cardiogenic Shock: A Nationwide Inpatient Study
Source: Clin Cardiol. 2026 Jul 23;49(7):e70416. doi: 10.1002/clc.70416 (PMC13392817; doi:10.1002/clc.70416)
Supplement: Supplementary file 1 — Supporting File [file CLC-49-e70416-s001.docx]

**Supplemental Table 1. Charlson-Adjusted Sensitivity Analysis of In-Hospital Outcomes by Mechanical Circulatory Support Strategy**

| Outcome / MCS Strategy | Ratio (95% CI) | P value |
| --- | --- | --- |
| **In-hospital Mortality*** |  |  |
| IABP Only | **0.58 (0.53–0.64)** | **<0.01** |
| ECMO Only | **2.41 (2.14–2.72)** | **<0.01** |
| Impella Only | **1.39 (1.24–1.57)** | **<0.01** |
| **Length of Stay (days)**** |  |  |
| IABP Only | **1.64 (1.57–1.70)** | **<0.01** |
| ECMO Only | **1.79 (1.68–1.91)** | **<0.01** |
| Impella Only | **1.19 (1.11–1.27)** | **<0.01** |
| **Estimated Hospital Cost, 2019 USD (USD)***** |  |  |
| IABP Only | **2.30 (2.21–2.39)** | **<0.01** |
| ECMO Only | **3.27 (3.10–3.46)** | **<0.01** |
| Impella Only | **2.30 (2.18–2.43)** | **<0.01** |

*Adjusted Odds Ratio for In-Hospital Mortality compared with No MCS

** Incidence rate ratios from negative binomial regression, representing ratio of expected LOS compared with No MCS.

*** Cost ratios from gamma regression with log link, representing ratio of expected cost (inflation-adjusted to 2019 US dollars) compared with No MCS.

Models adjusted for age, sex, race, ZIP income quartile, hospital teaching status, hospital region, urban-rural location, primary payer, and Charlson comorbidity index.

Bold indicates p<0.05.

**Supplemental Table 2. Predicted In-Hospital Mortality by Mechanical Circulatory Support Type**

| Model | None | IABP Only | ECMO Only | Impella Only |
| --- | --- | --- | --- | --- |
| **Elixhauser-adjusted Model** | 29.7% (28.8-30.6) | 19.9% (18.4-21.4) | 49.6% (46.8-52.4) | 36.8% (34.0-39.5) |
| **Charlson-adjusted Model** | 28.6% (27.9-29.3) | 19.0% (17.6-20.4) | 48.2% (45.5-50.9) | 35.4% (32.8-38.0) |

*Data are presented as percentage (95% confidence interval). Models adjusted for age, sex, race, ZIP income quartile, hospital teaching status, hospital region, urban-rural location, primary payer, and comorbidity index (Elixhauser/Charlson).


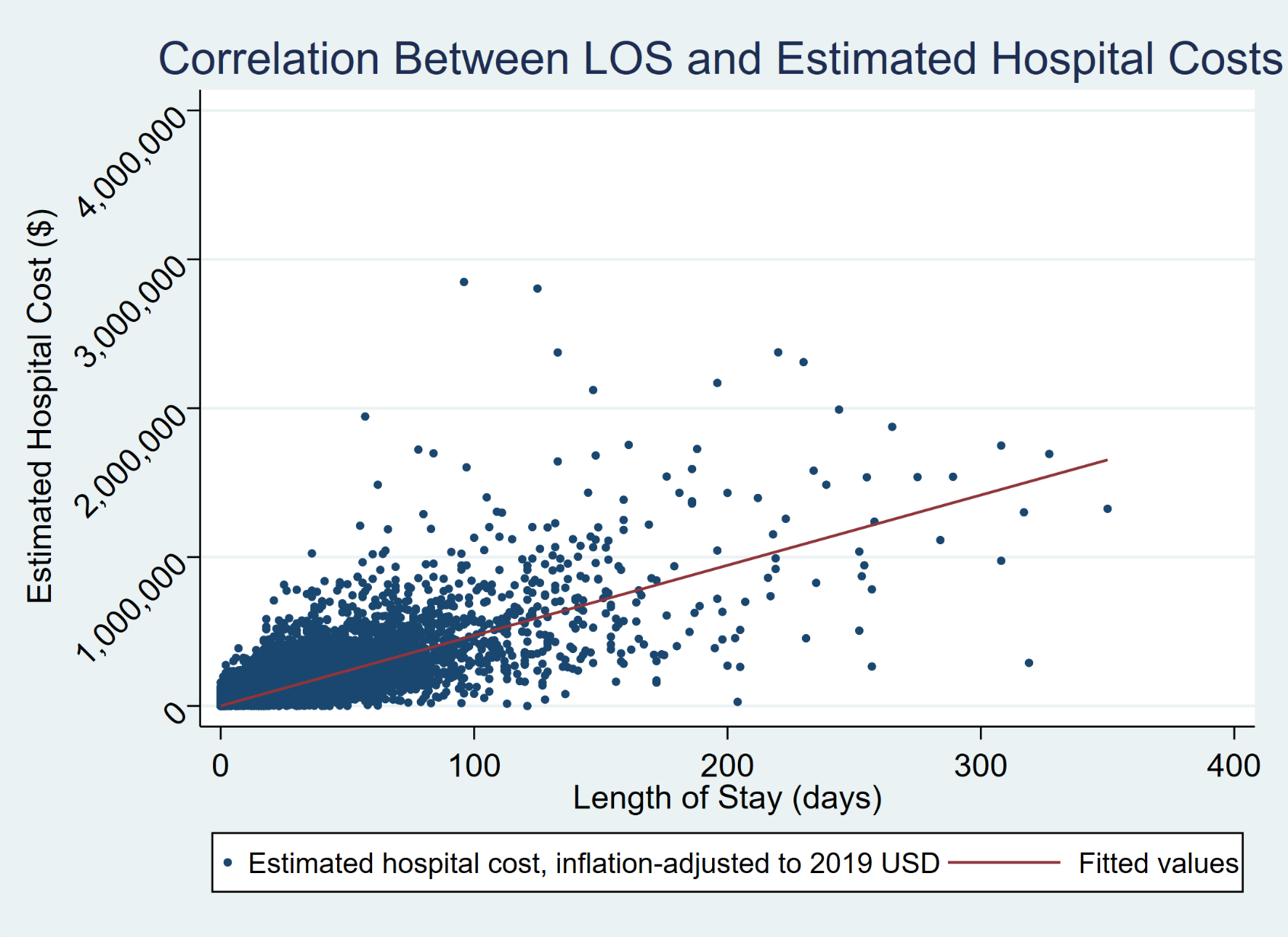


Supplemental Figure 1 Correlation between Total Cost of Hospitalization and Lengths of Stays
